# Evidence of Violations of the International Code of Marketing of Breast-Milk Substitutes since the Adoption by the World Health Assembly in 1981: A Systematic Scoping Review Protocol

**DOI:** 10.3390/ijerph18189523

**Published:** 2021-09-09

**Authors:** Genevieve E. Becker, Constance Ching, Paul Zambrano, Allison Burns, Jennifer Cashin, Roger Mathisen

**Affiliations:** 1 BEST Services, H91T22T Galway, Ireland; 2 Alive & Thrive Southeast Asia/FHI 360, Washington, DC 20009-5721, USA; cching@fhi360.org; 3 Alive & Thrive Southeast Asia/FHI 360, Manila 1101, Philippines; pzambrano@fhi360.org; 4 FHI 360, Durham, NC 27701, USA; aburns@fhi360.org; 5 Alive & Thrive Southeast Asia/FHI 360, Port Townsend, WA 98368, USA; jcashin@fhi360.org; 6 Alive & Thrive Southeast Asia/FHI 360, Hanoi 11022, Vietnam; rmathisen@fhi360.org

**Keywords:** scoping review, protocol, Code of Marketing, breast-milk substitutes, infant formula, marketing, child nutrition, breast-feeding, violation, compliance

## Abstract

This is the protocol for a scoping review that aims to systematically explore and summarise the published evidence of violations of the International Code of Marketing of Breast-milk Substitutes (the Code) and subsequent World Health Assembly Resolutions globally. The planned scoping review will seek to identify what research has been conducted on the topic, examine the geographic spread and nature of violations, and summarise knowledge gaps. The Code was adopted in 1981 by the World Health Assembly to protect infant health, in particular from aggressive and inappropriate marketing of breastmilk substitutes including formula and related products. Non-compliance with the Code or violations are described in reports, however, no existing systematic review of the global research appears to have been conducted that encompasses the varied disciplines including health, economics, and gender. The review will inform international and national decision-makers on the nature of violations and potentially highlight the need for new modalities to regulate this marketing. The proposed scoping review will use the six-step process of Arksey and O’Malley which includes defining the research question; identifying the relevant literature; selecting studies; charting the data; collating, summarising and reporting the findings; and will include a consultative group.

## 1. Introduction

Evidence of the importance of breastfeeding to health and non-health outcomes at individual and population levels has mounted over the past decade [1]. Not breastfeeding has negative effects on the health and wellbeing of the individual child and of the mother, on the human capital and economics of the family and of the nation, and environmental effects both locally and globally, and contributes to inequity and gender imbalance [2,3,4,5,6]. Despite this, only 44.4% of infants globally initiate breastfeeding within an hour of birth, only 42.2% are exclusively breastfed for six months, and only 43.9% continue breastfeeding for at least two years [7]. Breastfeeding is a collective social responsibility to create an enabling environment that removes barriers to enable its practice. One of the most potent barriers is the aggressive and inappropriate marketing of breastmilk substitutes (BMS) [8]. This marketing includes promotion to the general public, health workers and mothers directly and via media [9,10,11,12,13,14], donations of free and low-cost supplies of BMS and equipment to health services [8] and in emergency situations [15,16], gifts to health workers and to mothers [17,18,19,20], and unsubstantiated health claims [21,22,23,24], among other activities. The highly profitable marketing utilises highly effective strategies to influence the “first food system” to undermine breastfeeding, unduly influence the policy frameworks that protect breastfeeding from commercial influence, and ultimately influence the attitudes and practices of parents, caregivers, healthcare workers, even policymakers [25]. The COVID-19 pandemic was shown to be another platform and opportunity for harmful marketing of BMS and the undermining of breastfeeding [26].

As marketing increased in the 1970s, particularly in low- and middle-income countries, the increase in illness and deaths of children associated with formula feeding became a concern [27,28]. The International Code of Marketing of Breast-milk Substitutes (the Code) is an international health policy framework adopted by the World Health Assembly (WHA) in 1981 that aims to contribute “to the provision of safe and adequate nutrition for infants, by the protection and promotion of breastfeeding, and by ensuring the proper use of breast-milk substitutes, when these are necessary, on the basis of adequate information and through appropriate marketing and distribution” (Article 1) [29] “The Code” hereafter means the Code from 1981 and the subsequent relevant World Health Assembly resolutions to date. The Code [16] applies to all products that are marketed or otherwise represented as a suitable partial or total replacement of breastmilk, including any milk products marketed for feeding infants and young children up to three years of age; other foods and beverages marketed as suitable for feeding infants less than six months or for feeding from a bottle, and feeding bottles and teats. Pacifier use may be a risk factor for early breastfeeding cessation [30], however, their marketing is not covered currently by the International Code or subsequent WHA Resolutions though some individual countries may include it in their national legislation.

The Code is a legal instrument for governments to implement Article 24 of the Convention of the Rights of the Child (1989), the most widely ratified human rights convention [31]. Subsequent WHA Resolutions were passed to update the Code in the context of a changing marketing landscape, rapidly evolving industry practices, and in response to new scientific findings and recommendations on infant and young child feeding. [32]. WHA Resolution 69.9 on “*Ending inappropriate promotion of foods for infants and young children*” adopted in 2016 sought to clarify the scope of the Code to include products for children up to 36 months and provide guidance on preventing cross-promotion between complementary foods and BMS. Cross-promotion is a form of marketing promotion where similar packaging, labelling and branding tactics are used to promote another related product. In this context, it also prohibits companies using promotional activities of complementary foods and beverages to establish relationships with parents and caregivers to market BMS [33,34]. This subsequent resolution to the 1981 Code also sought to highlight and avoid conflicts of interest between the BMS companies and the health system, among others.

The Code is not only about protecting breastfeeding. Infants and young children who are not breastfeeding also need protection. Their families and the health workers who assist them should be able to acquire information and support on the use of required products that are accurate, independent, and evidence-based information free from the marketing of a product or brand. This information should include what is appropriate for the needs of the child at that age, how to use the product including the health hazards of unnecessary or improper use of the products, and the societal and financial implications involved (Article 4) [29].

As of 2020, forty years from the first adoption of the Code by the WHA, 70% of 194 World Health Organization (WHO) member states had enacted legal measures with provisions to implement the Code—a pre-requisite to enforcement. However, only 25 countries had measures substantially aligned with the Code, and 58 have no legal measures in place [35].

Monitoring compliance is a challenging aspect of Code enforcement. This may be due to gaps in the actual legislation, the lack of local coordination mechanisms, the political and economic influence of BMS companies, and deficiencies in the human, technical, and financial resources required by the relevant local authority to conduct monitoring. Historically, civil society has taken up an active role in monitoring compliance with and violations of the Code [36]. The International Baby Food Action Network International Code Documentation Centre (IBFAN-ICDC) continuously published periodic Code monitoring reports of violations globally starting in the 1980s [37]. Other civil society organisations such as Save the Children, Changing Markets Foundation, Helen Keller International, World Vision, and First Steps Nutrition Trust have all published reports on Code violations in the past decade focused on one or more countries [21,24,38,39,40]. However, these reports are predominately published from an advocacy standpoint to hold companies to account and use disparate methodologies that make it difficult to assess, systematically and reliably, the prevalence of Code violations at the global level. Some reports were disputed by the formula industry [41,42] In 2015, WHO and UNICEF convened the Network for Global Monitoring and Support for Implementation of the International Code of Marketing of Breast-milk Substitutes and subsequent relevant World Health Assembly Resolutions (NetCode) with the aim of strengthening national legislation and monitoring and enforcement mechanisms [43].

Research related to the Code appears in studies from health, sociology, marketing, gender, economics, trade, development, environment and other disciplines. This broad context indicates a need for a systematic scoping review that brings these findings from separate disciplines together. A preliminary search for existing scoping or systematic reviews of published research on violations of the Code indicated no existing reviews.

The aim of this scoping review is to systematically explore and summarise the published evidence of violations of the Code in worldwide settings, to identify what available research has been conducted on the topic, examine where, how and involving who and what products, and to identify knowledge gaps. The findings will provide an overview of the nature of violations, provide helpful insight to strengthen Code scope, adoption, implementation and monitoring, and potentially highlight the need for new modalities to regulate BMS marketing.

The review will be illustrative of the scope of Code violation and is not anticipated to be an exhaustive report of violations.

## 2. Materials and Methods

The format of a scoping review was chosen as the most appropriate method to explore a broad body of evidence, identify key concepts and themes, and clarify sources of evidence. A scoping review maps the evidence available in a systematic manner and supplies a knowledge synthesis including research gaps that may aid future research planning [44]. This is a rapid scoping review to be carried out over a period of 10 weeks as an element of a wider project. To produce information in a short period of time we will streamline components of the systematic review process by addressing a specific limited number of research questions, limiting literature searches, and using tight eligibility criteria while aiming to maintain transparency of reporting [45]. The review protocol conforms to the Preferred Reporting Items for Systematic Reviews and Meta-Analyses Protocols (PRISMA-P) statement [46,47] in the sections relevant to a scoping review (Appendix A Table A1). During the process of the review, any significant changes made to the protocol will be documented and reported in the review. The proposed scoping review will use the method framework of the Joanna Briggs Institute (JBI) Collaboration [48] and will report in accordance with the guidance provided in the Preferred Reporting Items for Systematic Reviews and Meta-analyses (PRISMA) extension for Scoping Reviews (PRISMA-ScR) [49].

The scoping method will facilitate the exploration of this broad topic using an iterative approach based on constructivist theories and allow reflection on the search strategy, flexibility and further refining of the research questions as our understanding of the topic evolves. Detailed quality appraisal of studies will not be conducted as this review aims to explore the general scope of research conducted in this field.

The six-step process of Arksey and O’Malley [50] will form the framework for the work, which includes to define the research question; identify relevant literature; select studies; chart the data; collate, summarise and report the findings; and include a consultative group.

### 2.1. Step 1: Define the Research Questions

The aim of this scoping review is to systematically explore and summarise the published evidence of violations of the International Code of Marketing of Breast-milk Substitutes (the Code). To this end, the proposed review will seek to answer the following questions:

1. How geographically widespread are Code violations?
2. When do the violations occur?
3. In which settings are the violations occurring?
4. What is the nature and diversity of Code violations?
5. Who is/are the target of Code violations?
6. What products are involved in the violations?
7. What types of published research examined Code violations?

### 2.2. Step 2: Identify Relevant Literature

In order to be as comprehensive as feasible in identifying studies that will help to answer the research questions, specific search strategies will be created by a specialist librarian with input from the other research team members and reviewed using the PRESS standard (Peer Review of Electronic Search Strategies) [51]. Studies will be considered with data collected or published from May 1981 (the date of the adoption of the Code by the World Health Assembly) until June 2021. There will be no limitations to geographical location, country income level, social or cultural group, language, setting in which the violation occurs, journal impact factor or format of publication.

We planned a scoping literature review searching the following electronic databases chosen as providing comprehensive coverage of health, economic, and social science sources: PubMed (including Medline), Embase, Scopus, LILACS, Web of Science Core Collection, Web of Science SciELO, Sociological Abstracts, Proquest Dissertations and Theses Global, Global Index Medicus, CNKI, Global Health, Cinahl, PsycInfo, Africa-Wide Information, Academic Search Premier, Bibliography of Asian Studies, Business Source Premier, EconLit, ERIC.

Analysis of keywords in known articles related to the topic was undertaken to develop search terms relevant to Code, BMS, violation/compliance, and marketing. (Appendix A Table A2) We piloted the search terms in PubMed and Scopus to check and refine the search terms. Outcomes of the searches will be recorded with the database, number of items found, and any adaptations made to the search strategy. Results will be imported into the Endnote reference management system with duplicate copies removed.

To access the reports from organisations that the research team has identified as closely involved with the Code we will search the websites of WHO, UNICEF, International Baby Food Action Network (IBFAN), Access to Nutrition Initiative (ATNI), Helen Keller International, FHI 360/Alive & Thrive, World Vision and Save the Children.

To ensure a comprehensive view, we will scan the reference lists of included studies or relevant reviews identified through the search. Evidence in languages other than English for which the abstract can be translated adequately using Google translate will be included in the search with the full text translated as needed. If due to time and resource limits translations cannot be obtained, a list of possibly relevant titles in other languages will be provided as an appendix.

We are conscious that older research reports may be less evident in electronic searches and will purposefully search for these reports using known reports from civil organisations, checking references and citations and contacting knowledgeable persons.

### 2.3. Step 3: Select Studies

In line with the JBI guidance [48], we define the unit of examination to be research studies. Evidence sources will be considered when there is a systematic or organised investigation of a topic including a research question or problem, stated method of inquiry, description of analysis method, and reporting of findings. Types of study design can include primary research studies including trials, before and after studies, intervention studies, prospective and retrospective cohort studies, observation studies and others available in journals, organisation reports, or academic theses. Studies that included an aspect of interest in their methods but did not report findings on this aspect will be included to give a comprehensive view of the work performed. Opinion and discussion papers, policies, guidelines, reviews and studies solely focused on the effects of violations will be excluded.

The concept of interest is violations of the International Code of Marketing of Breast-milk Substitutes and subsequent relevant WHA resolutions. Violations will be considered which are within the scope of the Code and relate to Information and Education, Promotion to General Public and Mothers, Health Care Systems, Donations in Emergencies, Health Workers, Marketing Personnel, Labelling, Conflicts of Interest, Cross-promotion. Fortified milk drinks for pregnant women or breastfeeding women are not currently within the scope of the Code however they are an increasing means of cross-promotion and therefore we will include them in this review. We will use the definitions of the WHA Resolution 69.9: Marketing means product promotion, distribution, selling, advertising, product public relations and information, and Promotion is broadly interpreted to include the communication of messages that are designed to persuade or encourage the purchase or consumption of a product or raise awareness of a brand [33]. The assumption is made that marketing to pregnant women and new mothers is also likely to reach their families, health workers who provide care for these women and the general public.

Studies will be considered that occur in any context including, but not limited to primary or secondary or tertiary or quaternary health care, public or private sectors, emergency or relief settings and/or directed at any group (pregnant women, new mothers, health workers (pre-service or in-service), decision-makers, general public, school children) and/or via any means (leaflets, media, gifts, free samples).

Eligibility for inclusion requires that all three criteria are met:

1. The document is a primary report of a systematic or organised investigation of a topic including a research question or problem, stated method of inquiry, description of analysis method, and reporting of findings, and
2. The document reports on the specific violation(s) of the Code (original articles or including its subsequent WHA resolutions), and
3. The document reports on one or more specific context(s), setting(s), or means of marketing.

All titles and abstracts found in the searches will be downloaded and duplicates removed by AB. Abstracts will be independently screened by two members of the research team (GB, PZ, CC, JC, and RM) against the predetermined inclusion criteria and the reasons for excluding evidence will be recorded (Appendix A Table A3). Full text will be obtained and reviewed for all items where there is any uncertainty regarding inclusion. Any disagreements on inclusion will be discussed between the two research team members screening and if unresolved will be reviewed by a third research team member and further discussed. For any study published in the last ten years, we will seek additional information from study researchers where necessary to resolve questions about inclusion. Questions on the screening process and recording tool will be jointly discussed by the research team to harmonise understanding.

The results of the screening stage will establish a list of items meeting the eligibility criteria for the charting of their data. EndNote and Excel spreadsheets will be used to manage records and data. The number of studies included and excluded at each stage will be presented in a PRISMA flow diagram [52].

### 2.4. Step 4: Chart the Data

In seeking to ensure a consistent approach, a data charting tool was prepared by the research team using headings related to the research questions and established from a preliminary review of known evidence (Appendix A Table A4). This tool was piloted on three studies of varied types gathered during the piloting of the search strategy which were independently reviewed and charted by all research team members who would be involved in data charting. Discussion was held to address any questions and to check for consistency and relevance to the research questions and aim of the scoping review. The charting tool was then refined as needed and prepared for use in a spreadsheet format. At the commencement of the data charting stage to facilitate consistency, the first five items charted will be reviewed by the research team together and any questions discussed and clarified.

Where no evidence is obtained for an a priori heading this will be marked as such on the chart. Post-hoc headings will be added if identified as important following screening of the studies. Full text will be obtained for all included evidence. Research team members (GB, PZ, CC, and JC) will independently review and chart their assigned studies and checked by a second research team member. Any disagreements on the data will be discussed between the two research team members charting that item and if unresolved will be reviewed by a third research team member and further discussed. For any study published in the last ten years, we will seek additional information or clarification from study researchers where necessary to resolve questions about the data.

Included articles which are from open access journals will be assessed to determine if it is a “predatory journal” by checking is the journal listed in DOAJ (Directory of Open Access Journals) and/or is a member of COPE (Committee On Publication Ethics), and if still unclear the journal website will be reviewed for characteristics of predatory journals. Source of funding for the research will be recorded when this is included in the published item. Articles considered to be from predatory journals or with commercial funding related to BMS will be marked in the data charting and analysis will be carried out both with and without those articles included. The presence of predatory articles and funding sources will be discussed where relevant [53].

### 2.5. Step 5: Collate, Summarise and Report the Findings

Firstly, the Excel spreadsheet will be used for the basic numerical analysis of the number of studies, publication type, study characteristics, and funding source, and findings will be presented in tabular format with simple descriptive statistics as relevant. Geographic occurrences will be shown on a world map.

Secondly, the findings will be summarised and reported as a thematic narrative summary linked to the research questions identifying patterns in the data. Particular attention will be given to the historical “storyline” of violations to build a rich picture of the topic which spans a forty-year period.

Thirdly, the research team will discuss the findings, the completeness and knowledge gaps, how the findings might contribute to strengthening the Code scope, and overall implementation including monitoring and government enforcement. The discussion section will include how this review could be used to inform international and national decision-makers and contribute to reducing harm to infants and young children and their mothers as well as benefitting the wider community.

### 2.6. Step 6: Consultation

Consultation will occur during the development of the protocol and search strategy and during the drafting of the review, with information scientists, key researchers, Code trainers and experts and persons involved in guidance and monitoring of Code implementation. Their input will inform and be integrated via discussions with the research team. This consultation will be held via email and teleconferencing as appropriate. This group will be consulted to prioritise research questions, plan the search strategy, and discuss data elements to be charted and key elements to report. We will circulate to the consultative group a bibliography of the proposed publications to be included to ensure key literature is not missed. We will request the consultative group to review our findings before publication towards producing a clear and transparent report mapping the extent, range and nature of the literature on Code violations. We will involve the group in the plan for dissemination and opportunities for knowledge transfer. All the members of the consultative group are often involved with discussion related to the Code as part of their routine employment activity and therefore participating in this consultative process does not require ethical review procedures. No inputs from the consultative group will be published in any way that is individually identifiable.

## 3. Conclusions

This is the protocol for a systematic scoping review. The review is strengthened by following a structured process with any amendments to this protocol described in the review and the reasons, and by the involvement of a consultative group. However, we acknowledge there are likely to be limitations to this review; with over 190 countries in the world, and our limited resources, we will not be able to find all the reports of studies undertaken by researchers, governments or others that examined incidences of violations of the Code of Marketing. The span of the review covers forty years and with the digital age and open access revolution, we might have a substantial bias towards publications from the last decade; by including key dates in relation to the Code and the dates for the studies this will assist in seeing patterns of compliance or violation as they evolved. Though the search strategy is broad and includes non-English studies there may be studies that were not published in a format picked up by the electronic databases and our focused searches. As this is a scoping review there will be no quality appraisal of the included studies and therefore the robustness of the individual study’s data cannot be confirmed. The findings of this systematic scoping review and their dissemination through open access publication and highlighted via social media will contribute to further discussion and activity on the topic, serve to inform international and national decision-makers, provide helpful insight to strengthen Code implementation, monitoring, and enforcement, and thus contribute to reducing harm to infants and young children and their mothers.

## Data Availability

This is a protocol, and no data were generated.

## Diversity, Equity and Inclusiveness Declaration

The terms “woman” and “mother” and “breastfeeding” are used throughout our paper. Individual parents and families may use different words and we respect their preferred terminology.

## Appendix A

**Table A1:** PRISMA-P (Preferred Reporting Items for Systematic review and Meta-Analysis Protocols) 2015 checklist: recommended items to address in a systematic review protocol—adapted for a scoping review.

Section and Topic	Item No	Checklist Item
ADMINISTRATIVE INFORMATION		
Title:		
Identification	1a	Identify the report as a protocol of a systematic review
Update	1b	If the protocol is for an update of a previous systematic review, identify as such. *not applicable*
Registration	2	If registered, provide the name of the registry and registration number
Authors:		
Contact	3a	Provide name, institutional affiliation, e-mail address of all protocol authors; provide physical mailing address of corresponding author
Contributions	3b	Describe contributions of protocol authors and identify the guarantor of the review
Amendments	4	If the protocol represents an amendment of a previously completed or published protocol, identify as such and list changes; otherwise, state plan for documenting important protocol amendments. *not applicable*
Support:		
Sources	5a	Indicate sources of financial or other support for the review
Sponsor	5b	Provide name for the review funder and/or sponsor
Role of sponsor or funder	5c	Describe roles of funder(s), sponsor(s), and/or institution(s), if any, in developing the protocol
INTRODUCTION		
Rationale	6	Describe the rationale for the review in the context of what is already known
Objectives	7	Provide an explicit statement of the question(s) the review will address with reference to participants, interventions, comparators, and outcomes (PICO), *adapted for a scoping review as PCC: Participants (research studies), Concept, Context.*
METHODS		
Eligibility criteria	8	Specify the study characteristics (such as *PCC*, study design, setting, time frame) and report characteristics (such as years considered, language, publication status) to be used as criteria for eligibility for the review
Information sources	9	Describe all intended information sources (such as electronic databases, contact with study authors, trial registers or other grey literature sources) with planned dates of coverage
Search strategy	10	Present draft of search strategy to be used for at least one electronic database, including planned limits, such that it could be repeated
Study records:		
Data management	11a	Describe the mechanism(s) that will be used to manage records and data throughout the review
Selection process	11b	State the process that will be used for selecting studies (such as two independent reviewers) through each phase of the review (that is, screening, eligibility and inclusion in meta-analysis)—*meta*-*analysis—not relevant to this scoping review*
Data collection process	11c	Describe planned method of extracting data from reports (such as piloting forms, completed independently, in duplicate), any processes for obtaining and confirming data from investigators
Data items	12	List and define all variables for which data will be sought (such as PICO items—*PCC used in this scoping review*, funding sources), any pre-planned data assumptions and simplifications
Outcomes and prioritisation	13	List and define all outcomes for which data will be sought, including prioritisation of main and additional outcomes, with rationale
Risk of bias in individual studies	14	Describe anticipated methods for assessing risk of bias of individual studies, including whether this will be performed at the outcome or study level, or both; state how this information will be used in data synthesis
Data synthesis	15a	Describe criteria under which study data will be quantitatively synthesised
	15b	If data are appropriate for quantitative synthesis, describe planned summary measures, methods of handling data and methods of combining data from studies, including any planned exploration of consistency—*not relevant to this scoping review*
	15c	Describe any proposed additional analyses (such as sensitivity or subgroup analyses, meta-regression)—*not relevant to this scoping review*
	15d	If quantitative synthesis is not appropriate, describe the type of summary planned
Meta-bias(es)	16	Specify any planned assessment of meta-bias(es) (such as publication bias across studies, selective reporting within studies)—*not relevant to this scoping review*
Confidence in cumulative evidence	17	Describe how the strength of the body of evidence will be assessed (such as GRADE)*—not relevant to this scoping review*
From: Shamseer L, Moher D, Clarke M, Ghersi D, Liberati A, Petticrew M, Shekelle P, Stewart L, PRISMA-P Group. Preferred reporting items for systematic review and meta-analysis protocols (PRISMA-P) 2015: elaboration and explanation. BMJ. 2015 Jan 2;349(jan02 1):g7647.		

**Table A2:** Search terms piloted on PubMed.

Database	PubMed
Date of Search	27 July 2021
Number of Results	135
Search Strategy	((“code”[Title/Abstract] OR “code”[Other Term]) AND (“infant formula”[MeSH Terms] OR “infant food”[MeSH Terms] OR “milk substitutes”[MeSH Terms] OR “milk, human”[MeSH Terms] OR “bottle feeding”[MeSH Terms] OR “breast feeding”[MeSH Terms] OR “child nutritional physiological phenomena”[MeSH Terms] OR “infant nutritional physiological phenomena”[MeSH Terms] OR “maternal nutritional physiological phenomena”[MeSH Terms] OR “breast-milk”[Title/Abstract] OR “breastmilk”[Title/Abstract] OR “breast-milk”[Title/Abstract] OR “milk substitute*”[Title/Abstract] OR “bottle feed*”[Title/Abstract] OR “bottle fed”[Title/Abstract] OR “bottlefeed*”[Title/Abstract] OR “bottlefed”[Title/Abstract] OR “breast feed*”[Title/Abstract] OR “breastfeed*”[Title/Abstract] OR “breastfed”[Title/Abstract] OR “breast fed”[Title/Abstract] OR “artificial feeding”[Title/Abstract] OR “complementary feeding*”[Title/Abstract] OR “complementary food*”[Title/Abstract] OR “supplemental feeding*”[Title/Abstract] OR “supplementary feeding*”[Title/Abstract] OR “follow-up formula”[Title/Abstract] OR “follow-on milk”[Title/Abstract] OR “follow-on formula”[Title/Abstract] OR “growing-up milk”[Title/Abstract] OR “growing-up milk”[Title/Abstract] OR “human milk”[Title/Abstract] OR “feeding bottle”[Title/Abstract] OR “feeding nipple”[Title/Abstract] OR “feeding teat”[Title/Abstract] OR “specialised formula”[Title/Abstract] OR “condensed milk”[Title/Abstract] OR “powdered milk”[Title/Abstract] OR “milk powder”[Title/Abstract] OR ((“newborn*”[Title/Abstract] OR “infant*”[Title/Abstract] OR “baby”[Title/Abstract] OR “babies”[Title/Abstract] OR “toddler*”[Title/Abstract] OR “young child*”[Title/Abstract] OR “preschool”[Title/Abstract] OR “pre-school”[Title/Abstract] OR “maternal”[Title/Abstract] OR “mother*”[Title/Abstract]) AND (“formula*”[Title/Abstract] OR “milk*”[Title/Abstract] OR “food*”[Title/Abstract] OR “nutrition”[Title/Abstract] OR “feeding”[Title/Abstract] OR “beverage*”[Title/Abstract]))) AND (“violat*”[Title/Abstract] OR “complian*”[Title/Abstract] OR “non complian*”[Title/Abstract] OR “inappropriate*”[Title/Abstract] OR “conflict of interest”[Title/Abstract] OR “conflicts of interest”[Title/Abstract] OR “breach*”[Title/Abstract] OR “aggressive”[Title/Abstract] OR “undermin*”[Title/Abstract] OR “claim*”[Title/Abstract] OR “donation*”[Title/Abstract] OR “sponsorship*”[Title/Abstract] OR “tactic*”[Title/Abstract] OR “cross-promot*”[Title/Abstract] OR “cross promot*”[Title/Abstract])) AND (1981/1/1:2021/7/15[pdat])

**Table A3:** Screening of items of evidence.

Person Screening:	Date screened:		
Citation details (e.g., author/s, date, title, journal, volume, issue, pages) If translated, what is the original language and how was translation performed: Source: (e.g., database search, cited in another document, from researcher or other person, web site …) Multiple report: Is this study also reported in another publication? Multiple reports of the same study will be combined as one item of evidence. Citation details of other publication(s):			
Eligibility Q 1. Is the document a primary report of an original systematic or organised investigation of a topic including a research question or problem, stated method of inquiry, description of analysis method, and reporting of findings? Opinion and discussion papers, policies, guidelines and reviews will be excluded. Studies solely focused on the effects of violations will be excluded.	Meets Criteria: Yes or Unclear > Go to next question	No > EXCLUDE, check references and end	Reason to Exclude
Eligibility Q 2. Does the document report on specific violations of the International Code of Marketing of Breast-Milk Substitutes and its subsequent resolutions? (If only original Code mark for inclusion)	Yes or Unclear > Go to next question	No > EXCLUDE and end	
Eligibility Q 3. Does the document report on one or more specific context(s), setting(s) or means of marketing?	Yes > Proceed to data charting	No > EXCLUDE	
Eligibility Conclusion	Include	Exclude	Unclear
*Only continue to data charting if the item of evidence meets for the three Inclusion criteria. If the eligibility is unclear read the full paper and discuss with another research team member(s) to come to a decision.* *If the document is to be excluded check if the individual studies reviewed/cited in it are relevant to this scoping review and add to the list for screening.*			

**Table A4:** Draft data charting tool (Spreadsheet will be used).

Person Charting:	Date Charted:
Citation details (e.g., author/s, date, title, journal, volume, issue, pages)	
SECTION A: STUDY DESIGN AND INFORMATION	
1. Where study is published (e.g., name of journal, organisation, etc.)	
2. Date of publication	
3. Source(s) of funding for the study	
4. Publication type	
5. Type of study and design	
6. Data collection method	
7. Data collection period	
8. Use of pre-existing survey tool	
9. Sample size (e.g., number of participants, sites, etc.)	
10. Sampling method	
11. Sample characteristics	
12. Geographical location: Region/Country (or Countries)/City/Other (Specify)	
SECTION B: PRODUCTS AND VIOLATION	
1. Product(s) being marketed	
2. Types of violations	
3. When violations happened/were documented	
4. Where marketing is occurring	
5. Who marketing is directed at	
6. How marketing is carried out	
7. Companies (and/or brands) reported in the study	
8. Details of costs of the marketing	
SECTION C: OTHER INFORMATION	
1. Further information needed from the study authors	
2. References noted for potential inclusion or background	
3. Additional details and notes	
